# Need to change present regulatory framework for blood banks in India

**DOI:** 10.4103/0973-6247.75962

**Published:** 2011-01

**Authors:** Nabajyoti Choudhury

**Affiliations:** *Editor, Asian Journal of Transfusion Science, India*

There is continuous debate among blood bank personnel whether blood bank should come under the purview of the regulatory authority overseeing Food and Drug Administration (FDA). We, blood bankers (*per se* transfusion medicine specialists), want to justify this fact that it should be out of FDA purview because blood is not a drug but liquid tissue. This is a true statement. However, we should remember that all blood and blood components have medicinal values. For example, we expect a rise of 1 g% of hemoglobin after transfusion of every unit of whole blood or red blood cell. That is why blood banks will come under the regulatory purview of FDA for years to come as per specific regulation decided by the law of the land. We can see the same law applicable in many countries in the world to regulate blood bank industry.

We will discuss about Drug Rules of Drugs and Cosmetics (DandC) Act (1940), the Indian regulation which governs blood banking system in India. As mentioned in the name of the Act, it was enacted in 1940 but there were multiple amendments in between to cope up with changes in the last 70 years. The government of India regulatory body is Central Drugs Standard Control Organization (CDSCO) which is headed by the Drugs Controller General (India). Every state government has state level regulatory bodies for implementation of regulation inside provincial boundaries. Indian regulators have many more areas to look after and one of them is blood banks. As per an announcement made in the website of CDSCO (www.cdsco.nic.in), there are about 2609 blood banks in India. The website was last updated in June 2009. As the number of blood banks in India is increasing gradually, the correct figure should have been more than 2700 by this time. As per the website, there are about 940 (39.4%) blood banks managed by the central or state government; 376 (14.4%) blood banks are voluntary in nature; 753 (28.8%) blood banks are private hospital based blood banks and 540 (20.7%) blood banks are designated as private charitable type.

All these blood banks are governed by DandC Act which needs urgent revision due to advancement in transfusion science and new practices coming into the routine blood bank management. There was recent attempt by the CDSCO in this direction and the draft was discussed among many experts at different state levels. Recommendations are also sent to the DCGI (I) by multiple groups. As per the available document from expert groups, proposed amendments need more discussions. As workers in the field of TM, we sincerely hope that this document should come out as a model document for the development of this speciality to the next generation. This document should serve as a model regulatory document for all Asian countries as there are many similarities in standards in this region.

Unfortunately, the first draft had many unnecessary inclusions or omissions which need immediate correction. Let us focus on a few important points in the draft. Clause 122-G states the qualification of the blood bank in-charge. It is encouraging that DNB (TM) is being included with MD (TM). We should encourage our new breed of TM experts. However, it should also be remembered that we have more than 2700 blood banks in India and the number is increasing by more than 30 per year (approximately) due to increased corporate hospital based blood banks, new medical colleges and also in other categories. Minimum requirement in previous version of the Act was a person with MBBS with 1 year experience in blood banks, is being totally eliminated for future working, except those who are enlisted. On the other hand, there are about 12 institutes offering post graduate degrees in the country and a maximum of about 15 TM specialists are coming out every year. And if 5% of blood bank officers are retired in a year, there will be at least deficit of sizable number (about 200) of blood bank officers. Qualified TM specialist may not like to serve in small cities due to remuneration or working conditions. It is felt that previous eligibility of MBBS should be retained but working experience should be increased from 1 year to about 5 years in a licensed blood bank with component facility, to head a blood bank. New regulation may also make it mandatory credit system for blood bank officers for attending CMEs and workshop to retain their affiliation with state FDA.

There are representations from some of our colleagues who may not be medical doctors but are sufficiently equipped with knowledge possessing PhD degree and working in Transfusion Medicine for many years. What is the “medical need” of a medical doctor in a blood bank? Arguably, it is to take care of whole blood and aphaeresis donors, which needs medical attention. However, a medical doctor can work in all sections of the blood banks. If you look at the management of the blood centers (Red Cross or Community) in western countries, they are usually headed by non-medical managers like pharmacist or MBA. They opine that blood banks are collection and production facilities which need good managers and people specialized in good manufacturing practices (GMP). Then, where do Transfusion Medicine specialists work? In many of western countries, they are the interphase between stand alone community blood banks and hospital based blood banks and they practise bed-side transfusion medicine. They are also involved in all other sections of blood banks including heading the centers. This concept is eventually going to come to our country in days to come. That is why new amendments may include PhD qualification for blood bank in-charge.

Major rethinking is necessary for proposed regulation under part XII-B (General and Accommodation). It is proposed to increase floor areas of blood banks as per collection of the blood bank. In this, an encouraging move is to regulate the working area in a blood bank. However, calculation of carpet area with blood collection seems to be over-calculated. The table shows a floor space requirement of 160 m^2^ for a blood bank collecting 5000 units per year which will need 1000 m^2^ when the blood bank increases collection to 20,000 units per year. It is known to us that space requirement does not go doubling or tripling with every 5000 units of collection. It depends upon efficiency of blood bank how it utilizes equipment and man-hour with increase of work load. This calculation is arbitrary and there is no literary support to justify this enormous space requirement. Moreover, costs on floor space in different cities are different and maintaining this high space requirement is unviable for many blood banks. We sincerely hope that CDSCO will examine this issue with scientific facts so that many blood banks do not face closure due to unscientific calculation.

Under the heading of personnel, it is observed that technologists are required to have qualification of PG diploma in Transfusion Technology or MLT degree in TM out of total of five qualifications mentioned. To the best of our knowledge, there are no such courses available in the country at present. It is generally felt that central and state governments should start more courses for technologists, which produce quality technologists and are recognized by FDA. We should not bring some imaginary qualifications in a federal regulation in presumption of starting the course in future.

In the proposed changes, manpower is categorically specified as per blood collection. It is a very good attempt so that the blood bank is not understaffed. If a blood bank collects more than 20,000 units per year, there is a requirement of seven “full-time” medical doctors as stated. In continuation of our previous discussion of qualification of doctors, it will not be possible for blood banks to get high number of medical officers (especially MD/DNB) to run blood banks. It may not be even possible in government medical colleges where doctors are mostly in rotation from the pathology department. We should remember that to work in a blood bank, all these doctors will have to include their names in the license which will eventually prevent rotation. Manpower planning should be logical as per workload and shift duties. We feel that at least certain number of doctors (after sufficient training) may be allowed to work on part-time basis, especially for blood collection and other non-emergency areas.

Other observations in the document force one to think that some amendments are proposed only keeping government medical colleges in mind. One is that resident doctors can attend blood donation camps. Few erroneous inclusions are observed in “general equipment and instrument”. Name of *thrombophobe ointment* should not be mentioned as it is a commercial name. Under mandatory equipment, requirement of incinerator should be removed as it is not allowed by the Ministry of Environment. Generators are shown as optional which should be made mandatory keeping in mind the erratic power supply in many parts of the country. Another interesting inclusion is that 80% of antisera should be purchased from the lowest price bidder (L1) and 20% from the next bidder (L2). This type of amendments should be an institutional issue and may not come in an Act. It is important to understand that when we sit is a higher government or other platform, we should oversee the interests of all concerned under the umbrella. Unfortunately, many a time, personal bias comes and individuals try to see which is applicable to their own institution and try to act accordingly.

In short, this is a very good and timely move by the CDSCO and NACO to update Drugs Rules of D&C Act (1940). It is the need of the hour and it will help us to establish model regulations for safe blood supply across the country. It is generally felt that few more areas should be covered under this regulation like nucleic acid testing (NAT), stem cell harvesting and processing, HLA testing, blood irradiation, etc. It should be discussed and debated for inclusion in the DandC Act.

All the concerned authorities should be careful not to roll out some Drug Rules which may force closure of many blood banks in India. However, we should also see that there is no compromise in achieving quality in blood banks. If some changes are aimed to reorganize blood banking in India like reducing number of stand-alone blood banks by bringing in stringent rules, it is improper and ill timed. We agree that there is a need to reduce number of blood banks or to reorganize them. But it is a long drawn process. First, we should make safe blood available in all corners of the country. Second, whatever blood and components are issued out of any blood bank, whether government, NGO or corporate, it should be made safe by implementing quality standards. Third, we need to change other rules (like Medical Council to have blood bank with every medical college) so that the growing number of new blood banks can be reduced. It is always better to have public debate among stakeholders before proceeding further. This draft document may be made available in the CDSCO website for transparency and knowledge of all blood bankers. As workers in blood banking system in India, we are waiting eagerly to see new vibrant Drug Rules under DandC Act which will ensure supply of safe blood at the right time in right quantity and to the right person.

